# Neutrophil extracellular traps formation: effect of *Leishmania major* promastigotes and salivary gland homogenates of *Phlebotomus papatasi* in human neutrophil culture

**DOI:** 10.1186/s12866-024-03270-z

**Published:** 2024-04-04

**Authors:** Fahimeh Firouzjaie, Niloofar Taghipour, Amir Ahmad Akhavan, Seyyed Javad Seyyed Tabaei, Soheila Rouhani, Maryam Shirazian, Ameneh Koochaki, Mahboubeh Fatemi, Nariman Mosaffa, Vahideh Moin Vaziri

**Affiliations:** 1 Department of Parasitology and Mycology, School of Medicine, Shahid Beheshti University of Medical Sciences, Tehran, Iran; 2 Medical Nanotechnology and Tissue Engineering Research Center, Shahid Beheshti University of Medical Sciences, Tehran, Iran; 3 Department of Tissue Engineering and Applied Cell Sciences, School of Advanced Technologies in Medicine, Shahid Beheshti University of Medical Sciences, Tehran, Iran; 4 Department of Vector Biology and Control of Diseases, School of Public Health, Tehran University of Medical Sciences, Tehran, Iran; 5 Department of Immunology, School of Medicine, Shahid Beheshti University of Medical Sciences, Tehran, Iran

**Keywords:** Neutrophil extracellular traps, *Leishmania major*, Salivary gland homogenates, *Phlebotomus papatasi*, Real time PCR

## Abstract

**Background:**

Leishmaniasis as a neglected tropical disease (NTD) is caused by the inoculation of *Leishmania* parasites via the bite of phlebotomine sand flies. After an infected bite, a series of innate and adaptive immune responses occurs, among which neutrophils can be mentioned as the initiators. Among the multiple functions of these fighting cells, neutrophil extracellular traps (NETs) were studied in the presence of *Leishmania major* promastigotes and salivary gland homogenates (SGH) of *Phlebotomus papatasi* alone, and in combination to mimic natural conditions of transmission.

**Material & methods:**

The effect of *L. major* and SGH on NETs formation was studied in three different groups: neutrophils + SGH (NS), neutrophils + *L. major* (NL), neutrophils + *L. major* + SGH (NLS) along with negative and positive controls in 2, 4 and 6 h post-incubation. Different microscopic methods were used to visualize NETs comprising: fluorescence microscopy by Acridine Orange/ Ethidium Bromide staining, optical microscopy by Giemsa staining and scanning electron microscopy. In addition, the expression level of three different genes NE, MPO and MMP9 was evaluated by Real-Time PCR.

**Results:**

All three microscopical methods revealed similar results, as in NS group, chromatin extrusion as a sign of NETosis, was not very evident in each three time points; but, in NL and especially NLS group, more NETosis was observed and the interaction between neutrophils and promastigotes in NL and also with saliva in NLS group, gradually increased over times. Real-time reveals that, the expression of MPO, NE and MMP9 genes increased during 2 and 4 h after exposure, and then decreased at 6 h in most groups.

**Conclusion:**

Hence, it was determined that the simultaneous presence of parasite and saliva in NLS group has a greater impact on the formation of NETs compared to NL and NS groups.

## Introduction

Leishmaniasis, is a complex group of neglected diseases, caused by the intracellular protozoan parasites of the genus *Leishmania*. The parasites are transmitted to mammals upon the blood meal of infected female phlebotomine sand flies and cause significant morbidity and mortality worldwide, including a wide range of the clinical manifestations of disease ranging from the self-healing skin lesions of cutaneous leishmaniasis (CL) to systemic involvement in visceral leishmaniasis (VL) which leads to death if left untreated. It affects over 150 million people worldwide with 350 million at risk in about 99 countries. Today, more than 1 billion people live in areas endemic to leishmaniasis and are at risk of infection. An estimated 30 000 new cases of VL and more than 1 million new cases of CL annually occur [1, 2]. The most common form is CL and in 2022, of the 200 countries and territories that reported to WHO, 99 were considered endemic for leishmaniasis. Out of them, 90 countries were considered endemic for CL, 80 countries for VL and 71 were endemic for both CL and VL. Eight countries comprising, Afghanistan, Algeria, Brazil, Colombia, Iran, Iraq, Peru and Syria, each reported > 5000 CL cases globally [2].

Despite the fact that CL is often not lethal, it is regarded as one of the health issues in endemic regions of Iran since it may cause malformed, chronic lesions and requires expensive, time-consuming treatments [3]. In some cases, the treatments that are commonly used are not efficient, with side effects and difficulty in accessibility. Moreover, drug resistance was reported against some of them. Besides, despite continuous efforts, there is no effective vaccine available. Thus, a better understanding of the immune response against the infection that is caused by *Leishmania* species or saliva of sand flies is required to better target immunomodulatory approaches [4, 5].

Initial events at the site of infection seem to be critical in the development of an effective, and protective immune response against *Leishmania* infection. Special attention was given to neutrophils, as these cells are the first cells that quickly and massively accumulate at the bite site [4, 6, 7]. Neutrophils are innate immune cells that migrate via the blood to the desired area in case of trauma or infection. These cells play a vital role in the elimination of many invading pathogens [4, 8, 9].

Neutrophils use several mechanisms to kill microorganisms, including producing ROS and the release of their toxic granule content in the local environment as degranulation or in the phagosome for phagocytosis. A special defense strategy in neutrophils that is called neutrophil extracellular traps (NETs), was described as a network of extracellular fibers consisting of DNA and histones and is decorated with various proteins/enzymes [4, 10, 11].

During NETosis, the permeabilization of the nuclear membrane increases and chromatin decondensation happens, which is dependent on the activity of neutrophil elastase (NE), myeloperoxidase (MPO) and peptidyl arginine deiminase 4 (PAD4) [11–14]. Following changes to the nucleus' shape and structure, DNA is released into the cytosol where it combines with cytoplasmic proteins like calprotectin or granular proteins such as cathepsin G, proteinase 3, lactoferrin, azurocidin, and matrix metalloproteinase 9 (MMP9). At last, the mixture is ejected outside from the cell [15].

It was shown that NETs are capable of arresting and removing a variety of microorganisms [10–12, 16] such as *Mycobacterium tuberculosis* [17], fungi [18, 19], HIV-1 [20], *Toxoplasma gondii* [21], *Eimeria bovis* [22], *Plasmodium falciparum* [23]. Also there are some researches about the formation of NETs when neutrophils encounter to different *Leishmania* species [24, 25]. But the remarkable point is in nature, the *Leishmania* parasite is transmitted biologically with the bite of a sand fly, whose saliva has pharmacological and immunomodulatory properties, and leads to inflammatory response, so it affects the host's immune system and by producing Th2 cytokines, it resulted in a more severe disease reflected by a larger lesions and higher parasite numbers, which is called the "enhancing effect" [26].

The role of saliva in modifying the host's immune response and providing a suitable environment for the establishment of the *Leishmania* parasite has been proven, however, its role in the formation of NETs is still unclear. There are rarely studies related to the effect of sand fly saliva on the formation of NETs alone and in combination with the *Leishmania* species.

In the present study the potential role of salivary gland homogenates (SGH) and *L. major* in NETs formation was evaluated by exposing the human neutrophils with *L. major* promastigotes, SGH and a combination of both (*L. major* + SGH). To achieve this goal, NETs formation was studied by three microscopical methods; also, the expression level of NE, MPO and MMP9 genes was evaluated by Real-Time PCR.

## Materials and methods

### Isolation of human neutrophils

Venous blood was collected from healthy adult volunteer under the following conditions; 1) age between 35 to 40, 2) no Chronic granulomatous disease (CGD) and underlying disease, 3) no history of travel to leishmaniasis endemic areas at least in one year prior to the sampling, 4) no smoking and alcohol drinking. To confirm that the volunteer possesses all including criteria, the complete blood count (CBC), differential count and NBT test were performed.

Neutrophils were isolated by dextran (Carl Roth, Cat No: 9004–54-0) density gradient sedimentation followed by centrifugation over Histoprep (BAG-diagnostics, Germany) to avoid RBC contamination. according to approved procedures [27]. Briefly heparinized blood was mixed with equal volumes of 3% dextran and was set upright at room temperature for 30 min. The leukocyte-rich plasma was centrifuged at 500 g and 4 °C for 10 min. The pellet was re-suspended in Hank’s buffer and was overlayed on Histoprep, then centrifuged at 500 g in room temperature for 30 min. To separate the RBCs, the pellet was re-suspended in sterile water for 28 s and immediately to restore tonicity; it was washed twice in sterile normal saline at 500 g and 4 °C for 5 min. Finally, purified neutrophils were re-suspended in RPMI 1640 (Biosera, France) medium and kept on ice until use. Cell viability of isolated neutrophils was monitored by trypan blue staining as 99% and purity was determined by Geimsa staining and cell differentiation as 98% and confirmed by flow cytometer.

### *Leishmania major* culture

*Leishmania major* promastigotes (MRHO/IR/75/ER) were obtained from the Department of Parasitology, Tehran University of Medical Sciences. Male BALB/c mice (6–8 weeks old), were purchased from Pasteur Institute of Iran at different time interval and maintained under conventional conditions in the animal care facility. They were infected by subcutaneous inoculation of *L. major* promastigotes (3 × 10^6^) at the base of the tail. After weeks, the ulcer appeared and about 2–4 weeks later, the mice were euthanized by cervical dislocation and the spleens were separated and briefly immersed in a penicillin–streptomycin solution and then washed with sterile PBS then were homogenized with a sterile cell strainer and cultivated in RPMI 1640 (Biosera, France) media supplemented with 15% of heat-inactivated fetal bovine serum (FBS) (Gibco, USA) and 1.5% of penicillin (100 U/mL), and streptomycin (100 μg/mL) (Gibco, USA) and 2 mM L-glutamine and was incubated at 25 °C to release the amastigotes and change to promastigotes. After 5 to 6 cultures, stationary-phase promastigotes were obtained.

### Salivary gland homogenate preparation

*Phlebotomus papatasi* sand flies (originating from Badrood rural district, Natanz, Esfahan Iran) were reared at phlebotomine sand fly insectary, School of Public Health, Tehran University of Medical Sciences, Tehran, Iran. Female sand flies (3 to 5-day-old/ unfed) were selected and were kept for 2–3 min at -20 °C to make them immobilized. The salivary glands were dissected using fine forceps and needles in cold phosphate-buffered saline (PBS; pH = 7.4) under a stereo microscope, and then transferred into 1.5 ml micro-tubes and stored in PBS at-20 °C till use. To prepare SGH, immediately before experiment, salivary glands were disrupted by ultra-sonication (Sonic-300, Artec, USA) in 200 µl cold PBS (5 cycle of 20 s with 1 min interval on the ice), then centrifuged at 4000 g for 15 min and supernatant is collected. The BCA Protein assay kit (SK3021, Canada) was used to measure the total protein of SGH, according to the brochure and the absorbance was measured at 562 nm on a plate reader against blank and was calculated based on the standard curve. The equivalent of a half-pair of salivary glands was used for each treatment.

### Study groups

The effect of *L. major* and SGH on NETs formation was investigated in different groups designated in Table 1.

**Table 1:** Definition of controls and treated groups

Groups	Definition
Negative control	Untreated neutrophils
Positive control	Neutrophils + Phorbol Myristate Acetate (PMA) (100 nM)
NS	Neutrophils + SGH
NL	Neutrophils + *L. major* promastigote
NLS	Neutrophils + *L. major* promastigote + SGH

It should be mentioned that for treat of each group, 1.5 × 10^6^ neutrophils, 7.5 × 10^6^ promastigotes of *L. major* and 0.5 µg/ml of SGH were used accordingly. This amount of saliva is relatively consistent with natural condition, as sand flies inoculate the contents of one salivary gland into the bite site.

### Methods for NETs visualization

Three different microscopic methods were used to visualize NETs as follow:

1. Fluorescence microscopy by Acridine Orange/ Ethidium Bromide staining (AO/EB):

Dual Acridine Orange/ Ethidium Bromide staining (AO/EB) visualized under a florescent microscope for evaluation of the live status of the cells. Neutrophils in NL group were incubated with *L. major* (1 neutrophil/5 promastigotes ratio), in NS group with SGH (0.5 µg/ml, as in nature) and finally in NLS group with *L. major* + SGH at 37 °C in a humidified incubator with 5% CO2.

The AO/EB dye was prepared by mixing of 100 μg/ml of each in PBS [28]. After 2, 4 and 6 h post incubation, ready to use AO/EB dye was added to the mixture of each group (50/2 ratio), then specimens were analyzed with fluorescence microscopy (Nikon, Japan).

2 Optical microscopy by Giemsa staining

The treatments of each group were done based on what stated above. At each time point, a drop of each tube suspension was smeared lightly, air-dried in RT and fixed with absolute methanol, then the slides were stained with Giemsa dye (10%) for 8–10 min and then images were taken in an optical microscopy (Nikon, Japan). The percentage of neutrophils with NETosis was determined.

3 Scanning electron microscopy (SEM)

Based on the fluorescence and optical microscopic observations, the incubation time for SEM was considered between 4 to 6 h (37 °C with 5% CO2). To prepare the sample, the suspension was collected, and then fixed with 2.5% glutaraldehyde in 0.1 M sodium cacodylate buffer (pH 7.2). Thereafter, samples were placed on a slide coated with poly-L-lysine, post-fixed with 1% osmium tetroxide and dehydrated with graded series of ethanol. Then the specimens were coated with gold and analyzed by SEM (FEI ESEM QUANTA 200).

### Gene expression

The expression of three different genes NE, MPO and MMP9 was evaluated by Real-Time PCR. As described, human neutrophils were incubated with *L. major*, SGH and *L.major* + SGH in each group accordingly. All cell groups were collected and centrifuged at 2, 4 and 6 h after incubation, then immediately placed in YTzol pure RNA solution (Yekta Tajhiz, Iran) and stored at − 80 °C until used for RNA extraction.

### RNA extraction, cDNA synthesis, quantitative real-time PCR

Total RNA was extracted by YTzol pure RNA solution according to the manufacturer’s instructions. The quality and concentration of extracted RNA were determined using Thermo Scientific™ NanoDrop™ 2000/2000c and for evaluation of RNA purity, a 260/280 was measured. The cDNA of target mRNAs in a total volume of 20 μl was synthesized from 200 ng of total RNA following manufacturer's instructions (Yekta Tajhiz Azma, Iran). The resulting cDNA is amplified using target-specific primers (Table 2) in a QPCR for quantification of mRNA transcription of NE, MPO and MMP9 genes. Reactions were performed in a final volume of 10 μl consist of 5 μl Real Q Plus 2 × Master Mix Green High ROX™ (Amplicon, Denmark), 10 μM of each primer pair, 1 μl of cDNA, and RNase free water. The Real-time PCR was performed on a StepOne Plus™ instrument, LightCycler® 96 System (Roche, Germany). The mixtures were incubated at 95 °C for 10 min followed by 50 cycles of 95 °C for 15 s and 60 °C for 25 s, 72 °C for 25 s and followed a melting curve for gene expression analysis. The GAPDH was used as housekeeping gene or internal control for normalization. The assays were performed in duplicate and the results were analyzed using REST 2009® software based on 2 ^–ΔΔct^ method.

**Table 2:** The sequences of gene expression primers used for Real-time PCR assay

Genes	Sequences of primers (5′-3′)	Ref
GAPDH	Forward: CCACTCCTCCACCTTTGACG Reverse: CCACCACCCTGTTGCTGTAG	[29–31]
MMP9	Forward: GCCACTACTGTGCCTTTGAGTC Reverse: CCCTCAGAGAATCGCCAGTACT	[32–34]
MPO	Forward: GAGCAGGACAAATACCGCACCA Reverse: AGAGAAGCCGTCCTCATACTCC	[35, 36]
NE	Forward: GGAGCCCATAACCTCTCGC Reverse: GAGCAAGTTTACGGGGTCGT	[37]

### Statistical analysis

All the tests were done in duplicate. After checking the normality of data by GraphPad Prism Software version 8.4.3, since the data was normal, the results were analyzed by one-way ANOVA.

## Results

### Fluorescence microscopy observation

**Fig. 1:**
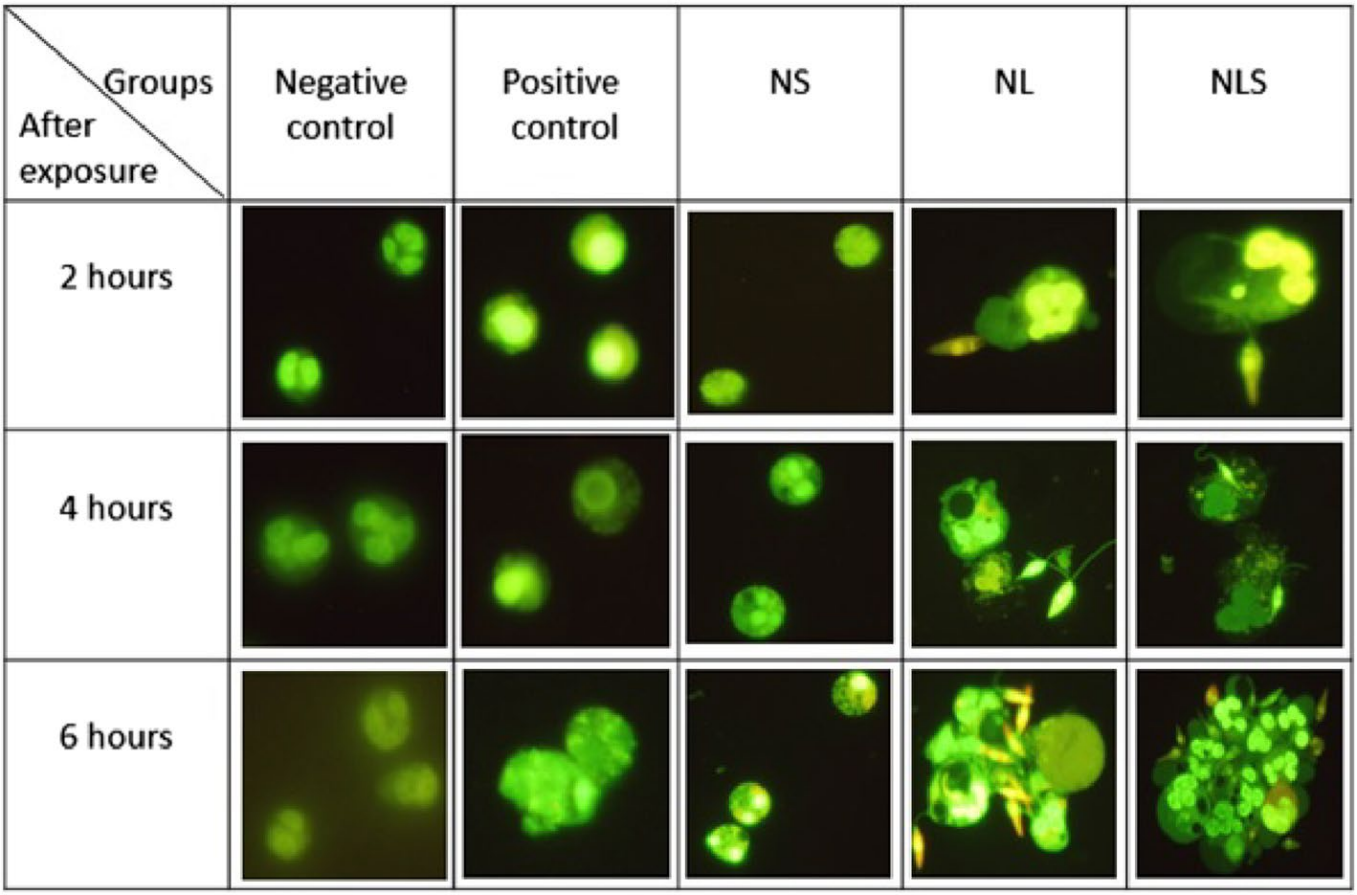
General overview of fluorescence microscopy using AO/EB staining to evaluate NETs formation. NS group showed less changes as NETosis compared to NL and NLS groups, however, the changes in NS distinguished it from negative control. In two other groups, particularly NLS, the interaction between neutrophils, parasites and SGH increased over time. (Original photo, magnification 400X)

**Fig. 2:**
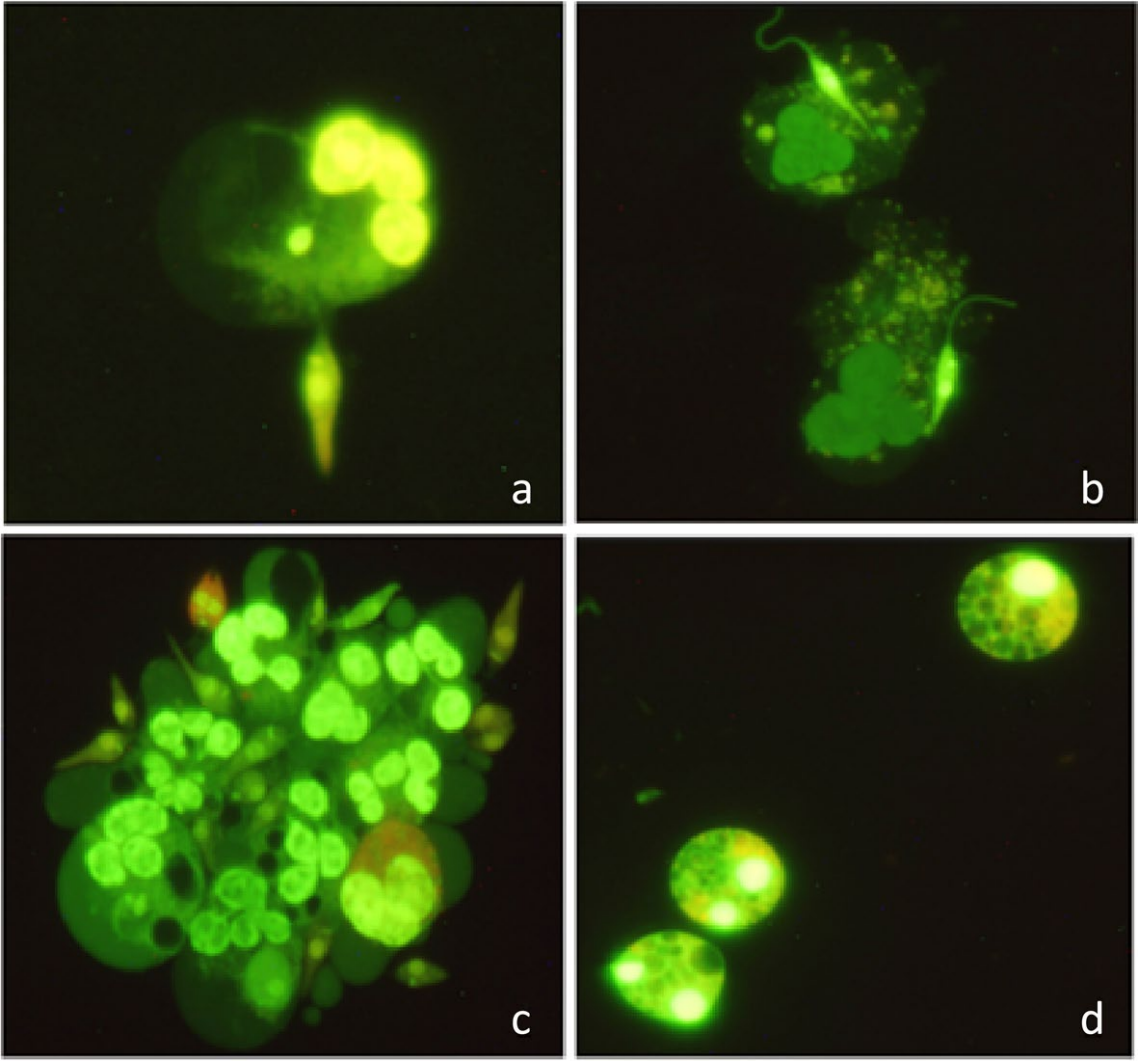
Fluorescence microscopy-based assessment of NETs formation. **a**, **b**, **c**) Neutrophil's interaction with promastigotes and SGH in NLS group respectively in 2, 4, 6 h post exposure **d**) Neutrophil intracellular changes in NS group 6 h post exposure (Original magnification 400X)

A general overview of the circumstances that happened to all treated groups in three-time points was shown in Fig. 1 and it was tried to depict the significant changes in Fig. 2. In NS group (Neu + SGH), although some changes were observable in each three time points compared to the negative control, chromatin extrusion as a sign of NETosis, was not very evident (Figs. 1 and 2d). But, in NL (Neu + *L.major*) and NLS (Neu + *L.major* + SGH) groups, two hours after exposure, the confrontation between neutrophils and promastigotes was one by one and a promastigote was arrested with a neutrophil (Figs. 1 and Fig. 2a). Furthermore, at 4 and 6 h later, this interaction increased and an accumulation of neutrophils and parasites was observed (Figs. 1 and 2b and c). This event was less in NL group and much more significant in NLS group (Fig. 1).

### Optical microscopy observations

**Fig.3:**
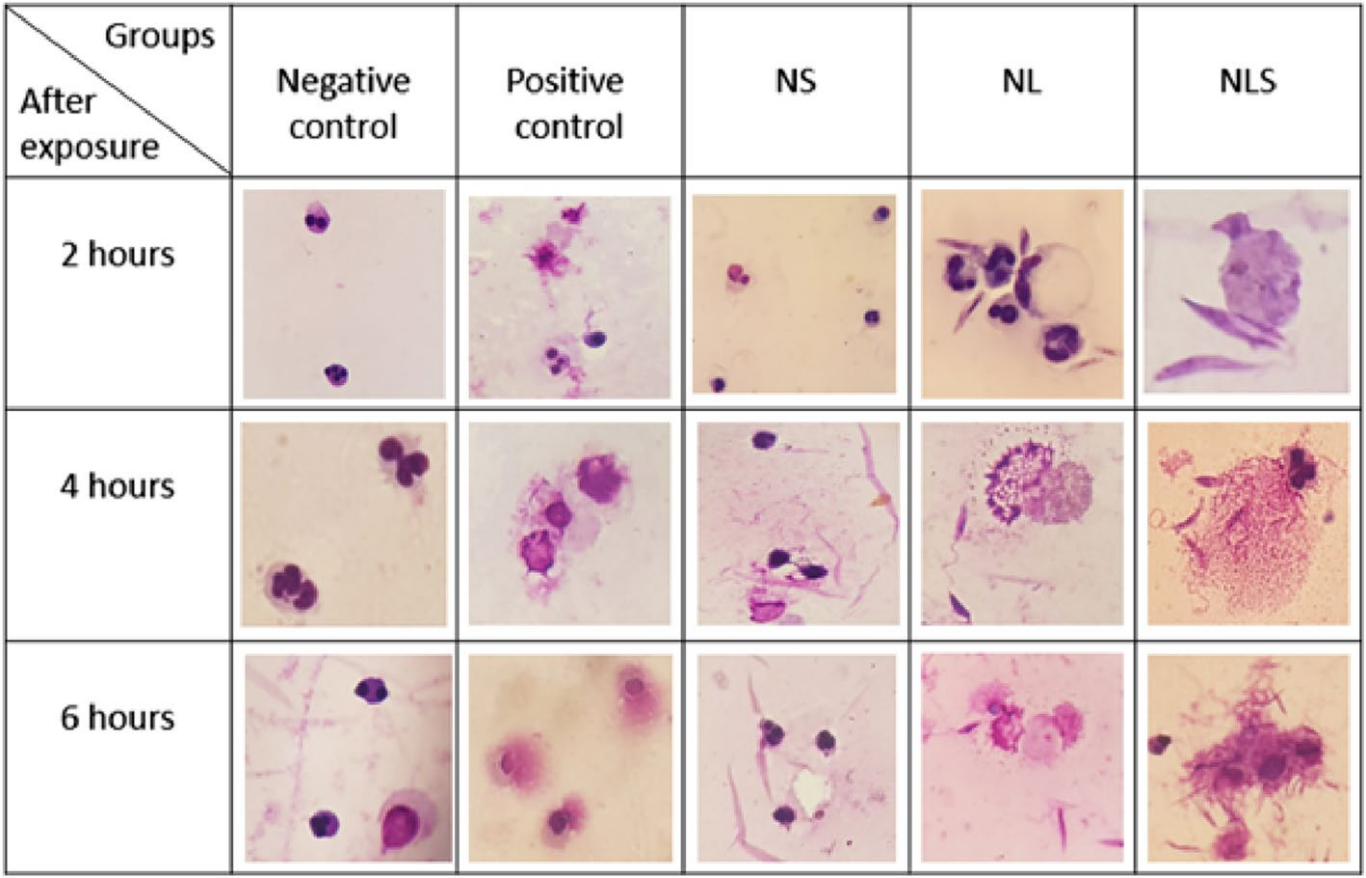
A comparative, general view of Giemsa staining among different groups (Images are captured with a 100 × magnification lens)

**Fig.4:**
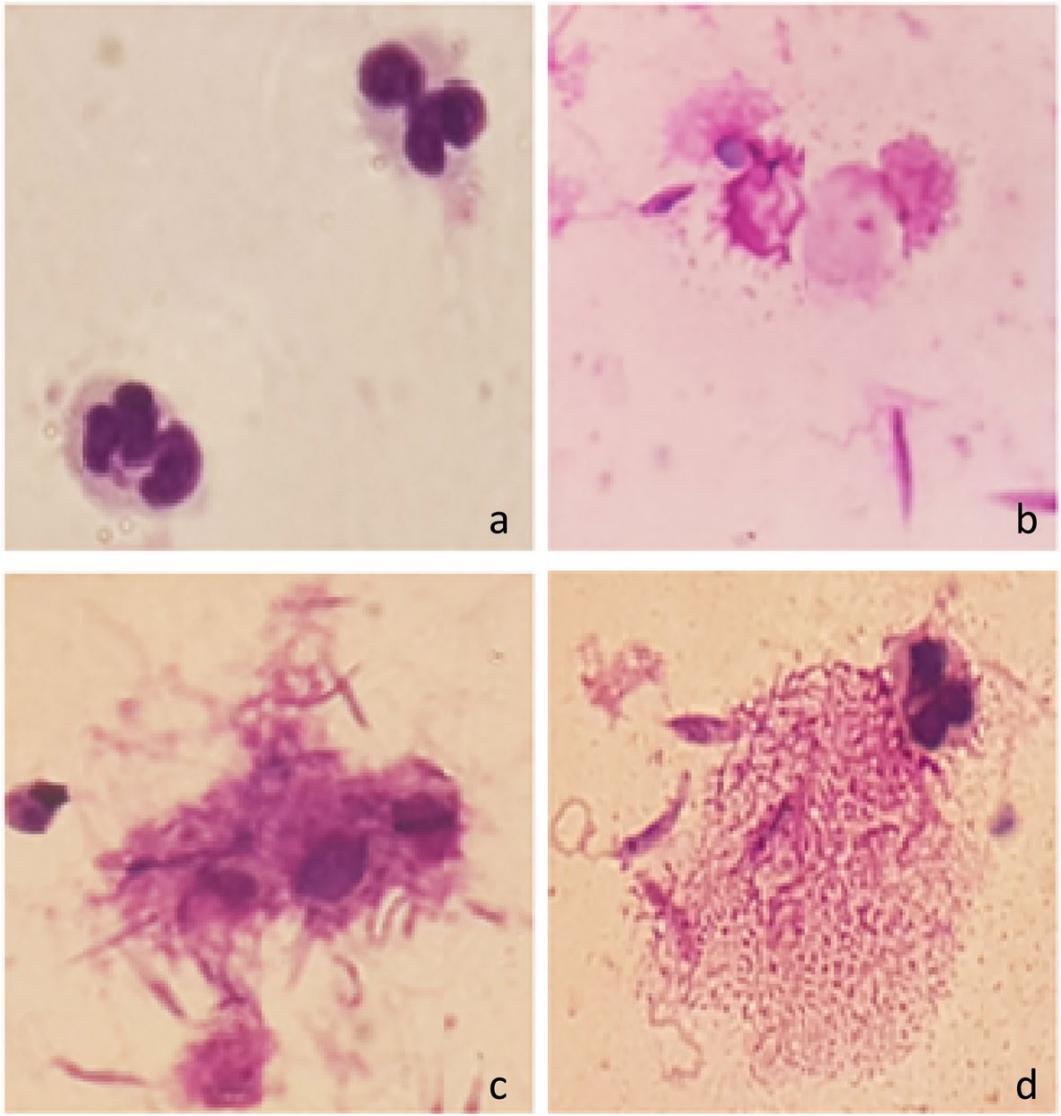
Giemsa staining of NETosis in NS, NL and NLS groups. **a** In NS group, no sign of NETosis was evident after 4 h post exposure in the most neutrophils, **b** & **c**) NETs formation 6 h after exposure in NL group and in NLS, respectively. **d** representative image of NETosis in NLS group 4 h after treatment

Along with the AO/EB staining method, the smears were prepared and stained by Giemsa; the general overview is shown in Fig. 3 which allows comparison between different groups, and the considerable changes was demonstrated in Fig. 4. It almost resembles the outcomes of the fluorescence microscopy technique. A small percentage of cells in NS group developed NETs, and apparently it was not much different from the negative control (Fig. 3 and 4a) But, in NL and especially NLS group, the interaction of neutrophils and promastigotes gradually increased over time and more neutrophils contributed to trapping the parasites during NETosis (Figs. 3 and 4b, c) (Table 3).

**Table 3:** The percentage of neutrophils under NETosis in NS, NL and NLS groups after different exposure times

Groups	NS	NL	NLS
2 h after exposure	8.8%	15.8%	17.5%
4 h after exposure	13.8%	18.3%	31.4%
6 h after exposure	17.6%	26.1%	38.8%

In NLS group, typical NETosis was observable and well-defined one was showed in Fig. 4d.

### Scanning electron microscopy

**Fig.5:**
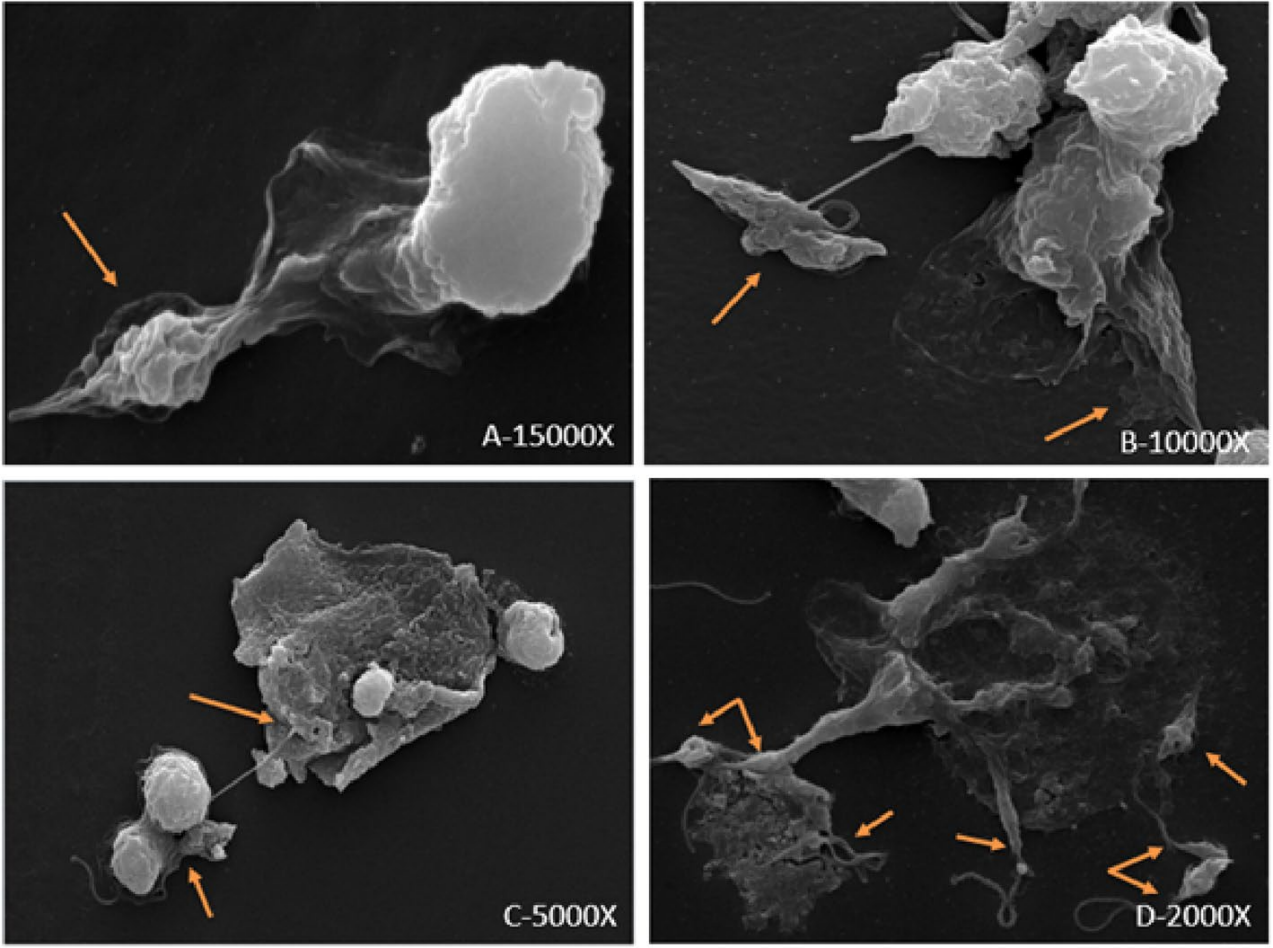
Scanning electron microscopy of different stages of NETosis in NLS group (4 h post exposure). **A**) Initial stage of NETosis in confrontation of neutrophil with parasite and SGH, **B** & **C**) progression of NETs formation and clear extrusion of chromatin, **D**) final stage of NETs, arrows indicate promastigotes

In SEM, similar to the staining methods, NETs formation was typical in NLS group and widespread NETs with trapped promastigotes were observed frequently (Fig. 5). It should be mentioned that, NETs distribution was not observed in NL and NS groups as it was in NLS.

### Expression level of NE, MPO and MMP9 genes

**Fig. 6:**
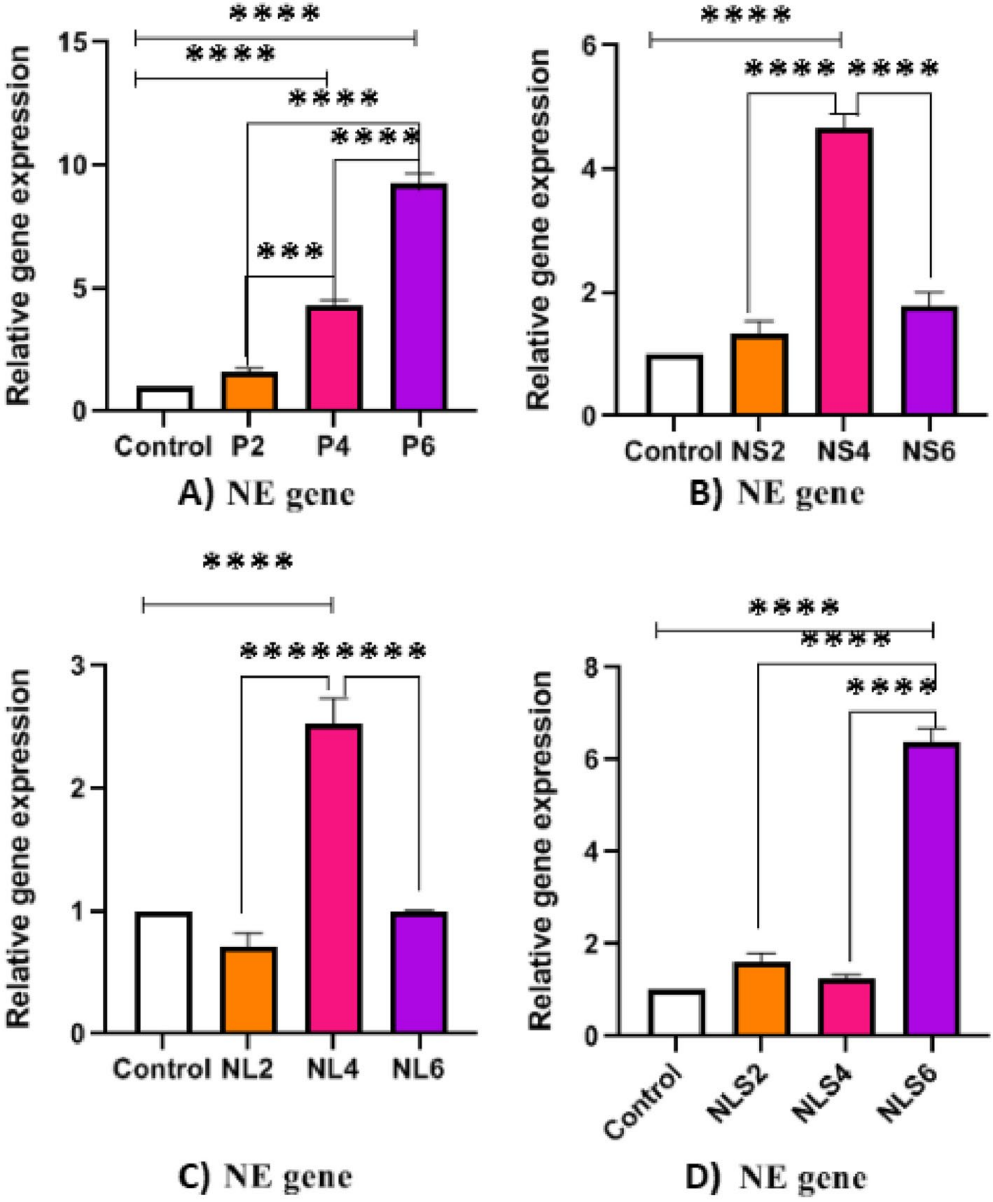
Comparative of Neutrophil Elastase (NE) gene expression between different groups in three time points **A**) Increasing level of NE gene expression in positive control group at 2, 4 and 6 h post exposure, **B** & **C**) Increasing level of NE gene expression at 2 and 4 and decreasing at 6 h post exposure in NS & NL groups respectively **D**) Elevating of NE gene expression at 2 h then decreasing at 4 h and increasing again at 6 h post exposure in NLS group, (*p* < 0.05 = *, *p* < 0.01 = **, *p* < 0.001 = ***, *p* < 0.0001 = ****)

**Fig. 7:**
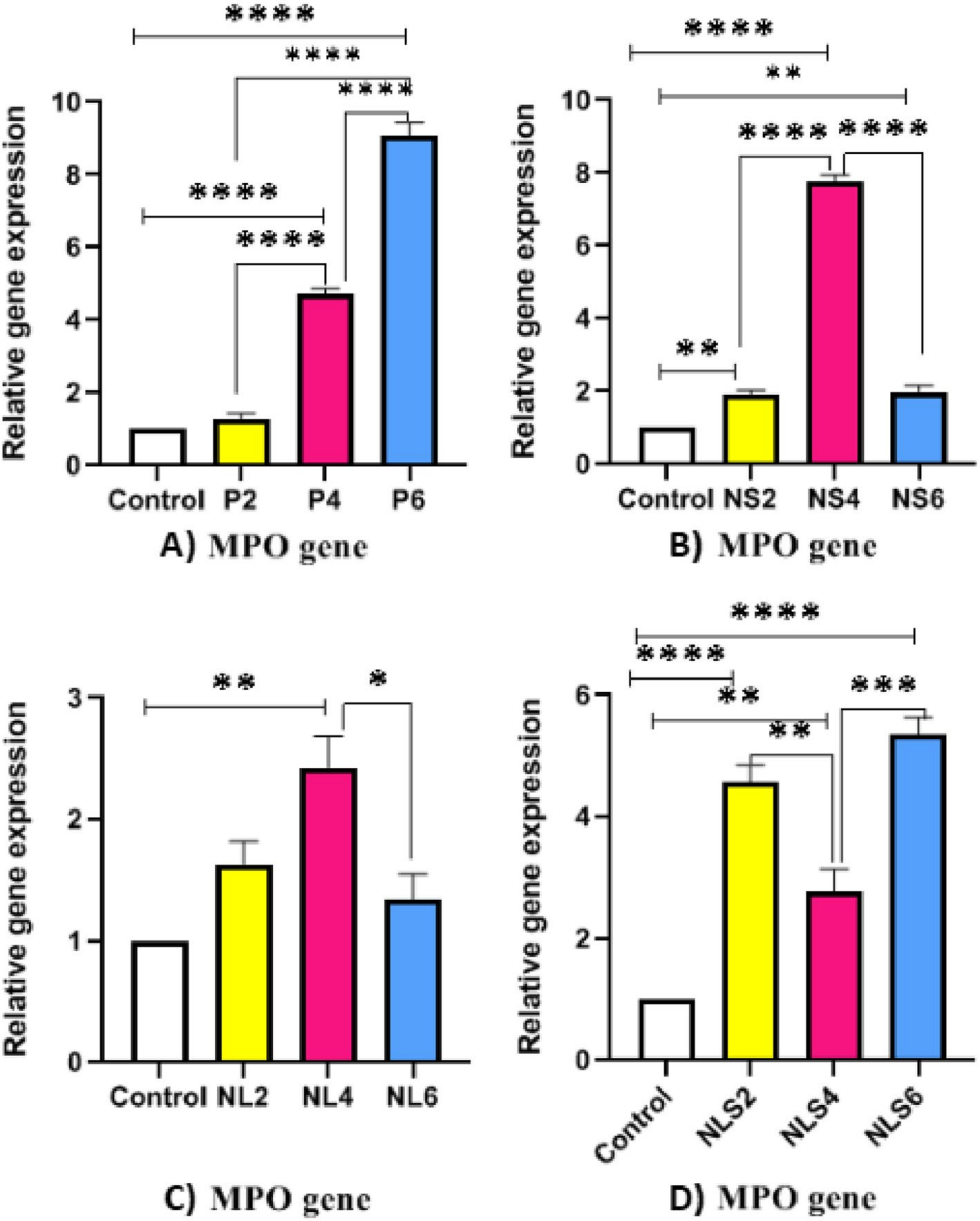
Comparative of Myeloperoxidase (MPO) gene expression between different groups in three time points **A**) Increasing level of MPO gene expression in positive control group at 2, 4 and 6 h post exposure, **B** & **C**) Increasing level of MPO gene expression at 2h and 4h and decreasing at 6 h post exposure in NS & NL groups respectively **D**) Elevating of MPO gene expression at 2h then decreasing at 4h and increasing again at 6 h post exposure in NLS group, (*p* < 0.05 = *, *p* < 0.01 = **, *p* < 0.001 = ***, *p* < 0.0001 = ****)

**Fig. 8:**
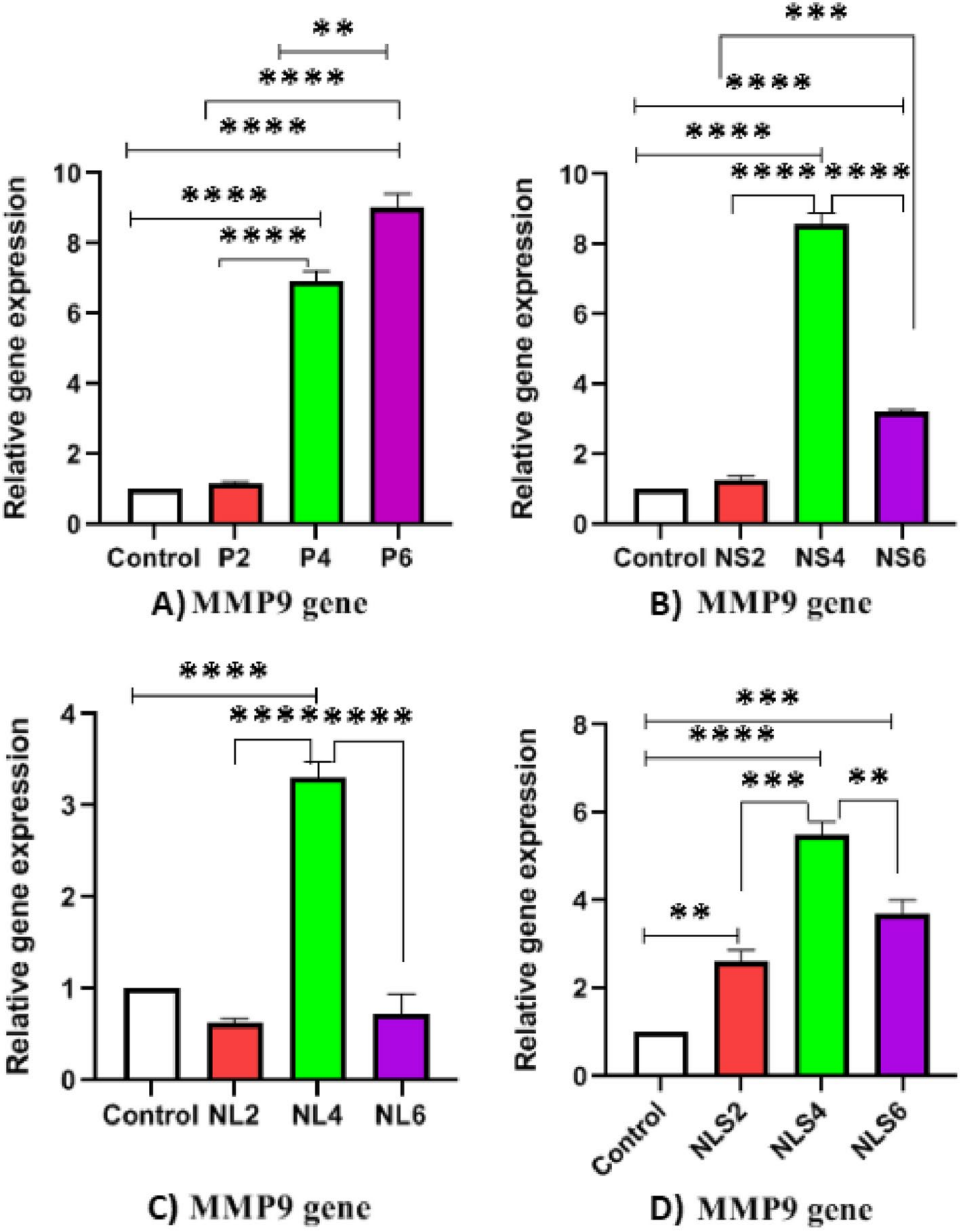
Comparative of Matrix Metalloproteinase 9 (MMP9) gene expression between different groups in three time points. **A**) Increasing level of MMP9 gene expression in positive control group at 2, 4 and 6 h post exposure, **B**, **C** & **D**) Increasing level of MMP9 gene expression at 2h and 4h and decreasing at 6 h post exposure in NS, NL & NLS groups respectively, (*p* < 0.05 = *, *p* < 0.01 = **, *p* < 0.001 = ***, *p* < 0.0001 = ****)

The expression of three different genes (NE, MPO and MMP9) was evaluated in all treated groups as well as in the control groups at 2, 4, and 6 h post-exposure. As expected, the expression of these genes increased over time in the positive control (Neu + PMA) (Figs. 6A, 7A, 8A). The trend of the expression level of NE, MPO and MMP9 was similar in all treated groups except in two, gene expression trend increased at 2, and 4 h after exposure and then decreased at 6 h post-exposure (Figs. 6B, C and Fig. 7B, C and Fig. 8B, C, D). In two exceptional groups that belong to NLS (Neu + *L.major* + SGH) the trend of MPO and NE expression was different. In these groups there was an increasing trend at 2 h, then a decrease at 4 h, and an elevation again at 6 h post exposure (Figs. 6D, Fig. 7D).

## Discussion

In leishmaniasis, after sand flies bite, a series of immune events involving innate and adaptive immune response occurs, among which, neutrophils can be mentioned as the initiators [38, 39]. Our objectives were to assess in vitro NETs formation on human neutrophils cultured with *L. major* and SGH of *Ph. papatasi* separately and together in three different groups so-called NS, NL and NLS.

Neutrophils are excellent at direct combat with wide effective functions, such as phagocytosis, production of reactive oxygen species (ROS), degranulation and formation of NETosis. So far, the mechanism of NETosis is less well-known mostly in terms of the short life span, inability to differentiate and lack of cell line for it. Because of this, there are still a lot of questions about the processes, nature, and products of NETosis, even after all that has previously been discussed. It is increasingly apparent how little is known about parasitic diseases and NETosis [40, 41]. Besides, the role of sand flies' saliva in NETosis formation, has been completely neglected in the existing limited studies on leishmaniasis. However, as far as authors know only one study was conducted in the new world on the impact of endonuclease LUNDEP in the escape of parasites from the function of NETs that lead to the exacerbation of *Leishmania* infection. Still there is a significant lack of this type of research in the old world [42]. Proteins of sand fly saliva are considered as neutrophil attractants and cause the accumulation of neutrophils at the bite site [43, 44]. On the other hand, it was shown that pharmacological and immunomodulatory properties of sand fly saliva induce an inflammatory response by the pathway of Th2 cytokine profiles that exacerbates *Leishmania* infection [26, 44]. Herein, an attempt was made to design and simulate a study similar to what happens in a natural *Leishmania* transmission. So, the effect of SGH on NETs formation alone and in combination with *L. major* was investigated for the first time.

It is noteworthy that the present study obtained similar outcomes through the utilization of three distinct microscopical techniques. Specifically, the concurrent existence of the parasite and saliva was found to have a more significant influence on the development of NETs in NLS group as opposed to NL and NS groups. It seems that SGH had a lower impact on inducing NETosis in comparison to *L. major* promastigotes. In a way, in NS group the observed morphological changes do not resemble what is expected in NETosis. While in NL and NLS groups more NETosis was observed, specifically in NLS group that simulates the natural condition of *Leishmania* transmission, significant changes were remarkable.

Further confirmation was done to validate these observations and the percentage of neutrophils under NETosis relative to the total number of neutrophils was calculated. Although the number of counted neutrophils was not enough for statistical analysis, it provides a rough estimation and confirms the microscopical findings. As far as the authors know there are no researches with the same goal or design of the current study, but it was proved that sand fly saliva facilitates the invasion of the parasite in the host by modulation of the immune system [45, 46].

Regarding the time points in our research, morphological changes and initiation of NETosis in NL and more especially in NLS group started two hours post-treatment. This phenomenon increased over time and the maximal NETosis was observed in NLS group after six hours of exposure. It was shown that most microorganisms, like *Leishmania* parasites induce classical or suicidal NETosis under in vitro conditions. This kind of NETosis starts 2 to 4 h after exposure and depends on ROS production of NADPH oxidase, besides neutrophil chromatin de-condensation mediated by MPO, NE, and PAD4 [41]. There is a study indicating that promastigotes of *L. amazonensis *are capable of inducing both classical and vital NETosis [47]. Early or vital NETosis occurs 5 to 15 up to 30 min after stimulation of neutrophils and it is independent of ROS production [41].

In this study, the expression of MPO, NE, and MMP9 genes was examined. After neutrophil activation, NE as a neutrophil serine protease, releases from azurophilic granules and moves towards the nucleus and there, partially degrades histones and promotes chromatin de-condensation. Thus, it is thought to be essential to start NETs creation [48]. Nonetheless, other research has produced contradictory findings, demonstrating that neutrophil serine proteases, such as NE, are not necessary for the production of NETs [49]. MPO is thought to work in concert with NE to promote chromatin decondensation [48]. Finally, MMP9 or gelatinase B, causes the accumulation of other neutrophils at the inflammation site and degrades the extracellular matrix by destroying gelatin and collagen, also causing tissue remodeling and​​ leading to apoptosis of endothelial cells by activating MMP2 [50, 51].

The current results showed that the expression of three mentioned genes increased in NS, NL and NLS groups during 2 and 4 h after exposure, and then decreased at 6 h. However, two exceptions in NLS group are notable and will be discussed further. The results showed that when SGH and promastigote of *L. major* were exposed alone, the expression of NE and MPO genes was increased after 4 h of exposure. The remarkable exception was observed in NLS group, in a way that, unlike the other groups, the expression of NE and MPO genes decreased at 4 h. This dissimilarity can probably be attributed to the inhibitory effect of saliva proteins on the host immune system, and its immunomodulatory settings in promoting the production of Th2 cytokines as has been shown in other studies [26]. This exception pattern was not observed in the expression of MMP9 gene in NLS group and the trend was the same as others (increasing at 2 and 4 h and decreasing at 6 h). This different expression pattern probably could be due to the functional time of these enzymes, as, NE and MPO are involved in the beginning of NETosis, but MMP9 releases later. It seems that the protein assay could provide better justification.

As evident in the obtained results, the highest expression level of NE, MPO and MMP9 genes was in NS group at 4 h post-exposure, while according to the microscopic observations minimal NETosis occurred in this group. This discrepancy could be explained by different neutrophil functions or by saliva properties in time-dependent manner.

Although during NETosis, the release of these enzymes as NETs features happens along with DNA strands into the extracellular environment, it should be noted that they are not exclusive to NETosis. Additionally, they are discharged during other neutrophil functions such as “degranulation” which occurs when neutrophils release their antimicrobial proteases that are enclosed in the shape of intracellular vesicles known as “granules” [52]. The choice of neutrophil functions is not well defined and it depends on environmental conditions, such as the metabolic changes, the size of the pathogen, the number of granules and age of neutrophils, etc. [53, 54].

On the other hand, the effect of arthropod's saliva on the accumulation of neutrophils was stated and interestingly the induction of neutrophil degranulation was proved after *Aedes aegypti* and *Hyalomma anatolicum* bites. Although it has not been proven for sand flies saliva but SGH may be capable of stimulating neutrophils towards degranulation rather than having the ability to induce NETosis [55, 56].

## Conclusion

The process of NETosis still has many unknowns, and here we have focused on a small piece of a large puzzle that occurs in innate and cellular immunity upon inoculation of the *Leishmania* parasite with saliva. We exposed *Leishmania* parasites and sand fly salivary glands to human neutrophils separately and simultaneously at different time points and evaluated the process of NETosis. It was confirmed that *Leishmania* parasites, along with sand fly saliva, have a higher potency to stimulate neutrophils towards NETosis. By the innovative approach of examining the combined effect of saliva and parasite on NETosis, it is believed that the present study's findings would open new avenues for applied scientific advancements and vaccine development. Exploring the potential effect of sand flies saliva on neutrophil degranulation, also investigations on other enzymes involving in NETosis like PAD4 or PADs family, cathepsin G, proteinase 3, lactoferrin and azurocidin, etc. could be helpful for more clarification of unknowns.

## Data Availability

No datasets were generated or analysed during the current study.

## Abbreviations

AO/EB: Acridine Orange/ Ethidium Bromide
BCA: Bicinchoninic Acid
CBC: Complete Blood Count
CL: Cutaneous Leishmaniasis
FBS: Fetal Bovine Serum
MMP9: Matrix Metalloproteinase 9
MPO: Myeloperoxidase
NE: Neutrophil Elastase
NETs: Neutrophil Extracellular Traps
NTD: Neglected Tropical Disease
PAD4: Peptidyl Arginine Deiminase 4
PBS: Phosphate-Buffered Saline
PMA: Phorbol Myristate Acetate
RBC: Red Blood Cell
SGH: Salivary Gland Homogenates
VL: Visceral Leishmaniasis
